# A Study of Mechanical Optimization Strategy for Cardiac Resynchronization Therapy Based on an Electromechanical Model

**DOI:** 10.1155/2012/948781

**Published:** 2012-10-16

**Authors:** Jianhong Dou, Ling Xia, Dongdong Deng, Yunliang Zang, Guofa Shou, Cesar Bustos, Weifeng Tu, Feng Liu, Stuart Crozier

**Affiliations:** ^1^Department of Anesthesiology, General Hospital of Guangzhou Military Command, Guangzhou 510010, China; ^2^Key Lab of Biomedical Engineering of Ministry of Education, Department of Biomedical Engineering, Zhejiang University, Hangzhou 310027, China; ^3^The School of Information Technology & Electrical Engineering, The University of Queensland, Brisbane, QLD 4072, Australia

## Abstract

An optimal electrode position and interventricular (VV) delay in cardiac resynchronization therapy (CRT) improves its success. However, the precise quantification of cardiac dyssynchrony and magnitude of resynchronization achieved by biventricular (BiV) pacing therapy with mechanical optimization strategies based on computational models remain scant. The maximum circumferential uniformity ratio estimate (CURE) was used here as mechanical optimization index, which was automatically computed for 6 different electrode positions based on a three-dimensional electromechanical canine model of heart failure (HF) caused by complete left bundle branch block (CLBBB). VV delay timing was adjusted accordingly. The heart excitation propagation was simulated with a monodomain model. The quantification of mechanical intra- and interventricular asynchrony was then investigated with eight-node isoparametric element method. The results showed that (i) the optimal pacing location from maximal CURE of 0.8516 was found at the left ventricle (LV) lateral wall near the equator site with a VV delay of 60 ms, in accordance with current clinical studies, (ii) compared with electrical optimization strategy of *E*_RMS_, the LV synchronous contraction and the hemodynamics improved more with mechanical optimization strategy. Therefore, measures of mechanical dyssynchrony improve the sensitivity and specificity of predicting responders more. The model was subject to validation in future clinical studies.

## 1. Introduction

Congestive heart failure (CHF) is a common heart disease caused by dilated cardiomyopathy or damage to the excitation conduction system. Biventricular pacing (BiVP), or cardiac resynchronization therapy (CRT), was a major breakthrough in the treatment for patients with advanced CHF complicated by discoordinate contractions caused by inter- or intraventricular conduction delays [1]. CRT has the potential to improve the quality of life and functional capacity, promote left ventricle (LV) reverse remodeling, and reduce heart failure (HF) hospitalizations and mortality in patients with New York Heart Association class III or IV CHF [2–6]. However, up to 20–30% of patients do not respond favourably to CRT using standard clinical selection criteria [7], and a critical analysis of the data suggests the true nonresponder rate can be estimated at perhaps 40–50% [8]. Data indicate that factors associated with a poor outcome are multifactorial and that inappropriate patient selection, left ventricular pacing site, and inadequate device programming are likely to be important [9]. Therefore, an individual optimal electrode position, atrio-ventricular delay (AV delay, AVD), or interventricular delay (VV delay, VVD) in CRT are needed to improve its success [10, 11]. However, left ventricular lead placement and timing delays continue to pose a number of challenges regarding the delivery of an effective CRT.

Over the past decade, many studies have examined the pathophysiology of cardiac dyssynchrony and tested the effect of cardiac resynchronization on heart functions and the efficacy of symptoms enhancements. Most of these researches have focused on the ECG characteristic of distinct responders versus nonresponders to CRT. For example, left and right ventricular leads were placed in different locations to attain the shortest QRS duration during biventricular stimulation. Presently, the optimal electrode setups and timing delays are also determined by measuring the cardiac index using magnetic resonance tagging (MR tagging), nuclear imaging (NI) or Doppler flow imaging and so on [12–15], most of which relate to the measurement of interventricular asynchrony (interVA) or intraventricular asynchrony (intraVA). 

However, it may be not sufficient for clinicians to formulate a scientific and reasonable treatment scheme for CRT optimization with only physical examination of ECG or medical imaging. Quantitative information and precise programming of the optimal stimulation delays and lead positioning accordant with the physiological function are needed. For example, although it is generally accepted that the posterior-lateral wall is the preferred location for LV lead location in CRT, other LV pacing sites have also been shown to offer superior benefits for selected patient population. In other words, there is no universally accepted best LV pacing site, nor the best V-V interval. Therefore, patient-specific CRT optimization is needed. There are both intersubject variability and intrasubject variability (e.g., compare resting versus exercise conditions). Modeling of heart can provide a useful supplementary tool to investigate the dynamical behaviour of heart quantitatively and thus enhance our understanding of the cardiac electrophysiological and mechanical properties under physical and pathological conditions. Consequently, the computer modeling of a patient-specific heart appears to be a reasonable solution for solving this problem, which can determine the optimal AV and VV timing pre- or postoperatively with respect to the lead positions in patients undergoing CRT. 

Many scientists have concentrated on the study of individual optimization of lead position and pacing delays of CRT by means of heart modeling and simulation with the progress of mathematical modeling techniques. The electrical-based optimization approach, for example, minimizing the QRS duration, has been widely accepted due to its simplicity. Therefore, most of researches have concentrated on the electrical strategies of optimizing parameters in CRT. Reumann et al. simulated an atrio-ventricular (AV) and a left bundle branch block (LBBB) with different reductions in the interventricular conduction velocity based on computer models of the Visible Man and a patient's heart [16]. They assumed that the ideal cardiac function is achieved if the excitation of the ventricles is as close as possible to the physiological rhythm. Consequently, the minimum error between the physiological excitation and pathology/therapy, that is, the root mean square error *E*_RMS_, was used as an optimization assessment index and automatically computed for 12 different electrode positions with different AV and VV intervals. Their results showed the importance of individually adjusting the electrode position, beside the timing delays, to the patient's anatomy and pathology in accordance with current clinical studies. Mohindra et al. recorded 120-electrode body-surface potential mapping (BSPM) data and calculated the epicardial potentials and isochrones of activation for different V-V intervals by means of an electrocardiographic inverse solution based on a ventricular computer model [17]. They used the area between the LV and RV percentage surface-activated curves as a measure of interventricular synchrony for a variety of V-V settings. Their results demonstrated that an optimal CRT pacing V-V interval can be selected by aiming to minimize the dyssynchrony in the ventricular activation patterns. However, they found that it was difficult to correlate the heart model results with the clinical recordings and moreover, the BSPM did not provide any information about how these patterns improved the cardiac output. To some extent, these mathematical models carried out well the adjustment of the optimization parameters of CRT. Yet, because the optimization procedure was based on the electrical criterion that was not necessarily equal to the electromechanical coupling [15], the simulations in these works might be wrong if the mechanical activation followed the electrical activation with an unfixed delay, that is,the electromechanical dissociation in the cardiopulmonary resuscitation (CPR) [18]. Thus, we doubt the soundness of the electrical optimization strategy for predicting effect of CRT. A relationship between electrical dyssynchrony and mechanical performance needs to be established. Nowadays, the importance of correcting mechanical dyssynchrony has been more and more realized in the field of CRT optimization. For example, most echo-based CRT optimization approaches target the mechanical synchrony, not the electrical synchrony. Unfortunately, the mechanical optimization strategy for CRT has seldom been investigated in the previous computational models.

Compared with previous electrophysiological models for CRT optimization, the present heart model in this paper is extended to include a mechanical contraction model for the optimization of the pacing lead locations and the interventricular delays (VVds) of CRT. Since the spatial distribution of local wall mechanics was very sensitive to the choice of myofiber orientations, specific data of real fiber orientations were used for true description of cardiac deformation in this study. The ventricular mechanical dyssynchrony is evaluated by the assessment of a circumferential uniformity ratio estimate (CURE) [15, 19]. The aim of this study is to validate the feasibility of CRT optimization with mechanical predictors (CURE) and to make a contrast analysis of cardiac function improvement between mechanical (CURE) and electrical (*E*_RMS_) optimization strategy for CRT. The present study resolves this question through the simulation of the cardiac electrical activation spread and the mechanical strain maps based on our previous electromechanical canine model of HF combined with a complete LBBB (CLBBB) [20]. 

## 2. Materials and Method

### 2.1. Heart Model of CHF with CLBBB

#### 2.1.1. Heart Anatomical Model

**Figure 1:**
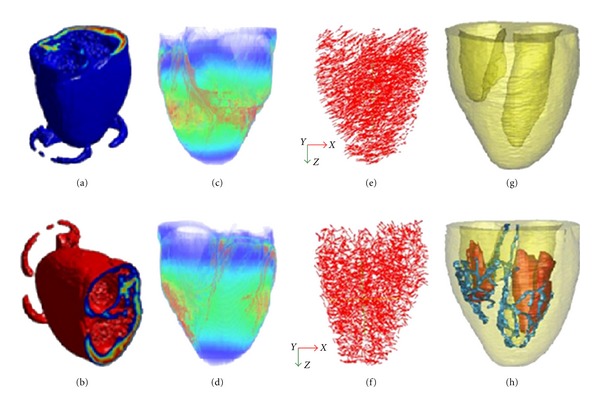
3D view of the canine ventricular anatomical model. The left panel ((a), (b)) shows anatomical geometry from original datasets. (c) and (d) display 3D view of the whole ventricles scanned from DT-MRI at Duke University Center visualized by Volview 2.0. (e) and (f) display distribution of 3-D fiber angles in the space. (g) and (h) display the reconstructed canine heart model.

The anatomical canine heart model used in this study was reconstructed from the MR scans of an intact dog heart derived at the Duke University Medical Center for In Vivo Microscopy, Durham, NC, USA (Figures 1(a), 1(b), 1(c), and 1(d)). The spatial discrete matrix size of the original data was 256 × 128 × 128, corresponding to a pixel size of 0.39 × 0.78 × 0.78 mm. Myocardial fiber orientations were obtained from diffusion tensor MR imaging (DT-MRI) in a 7.1 T MRI scanner (Figures 1(e), 1(f)) and different tissues were manually segmented into functional modules, including papillary muscles and Purkinje fiber networks (Figures 1(g), 1(h)). Full details of this model can be found in [20]. Note that papillary muscles are not used in the simulation.

#### 2.1.2. Heart Electrophysiological Properties

**Figure 2:**
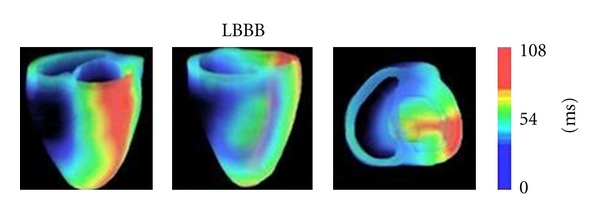
3D maps of simulated complete LBBB activation of the canine ventricles in different views.

To model electrical propagation in a dilated failing heart with CLBBB, an initial stimulus current with a magnitude of 100 *μ*A/cm^2^ and duration of 0.5 ms was added to the right bundle branch while blocking the conduction of the left bundle branch, leaving the rest of the conduction tree intact. Latest depolarization time in LBBB simulations occurred at 108 ms (see Figure 2). After these cardiac electrical excitation sequences were obtained, the active forces of myocardium can be calculated.

**(1) Ventricular Cell Model**

The dynamic ionic cell model developed by Winslow et al. [21] was used to simulate the electrophysiology of a single ventricular cell. The cell model with different ionic channel currents could be described by the following differential equations:

(1) $$\begin{array}{c} \frac{dV_{m}}{dt}=-\frac{1}{C_{m}}\left(\sum I_{\text{ion}}+I_{\text{app}}\right),\\ I_{\text{ion}}=G_{\text{ion}}\cdot \left(V_{m}-E_{\text{ion}}\right), \end{array}$$

where *V*_*m*_ is the transmembrane potential, *C*_*m*_ is the membrane capacitance, *I*_*x*_ is the current flow through the ion channel *x*,  *G*_*x*_ is the conductance of the channel, and *E*_*x*_ is the reversal potential for the channel [20].

**(2) Reaction-Diffusion Equations**

The asynchronous excitation propagation and intraventricular conduction in the LBBB were simulated based on solutions of reaction-diffusion equations of the monodomain model as shown in the following with a strategy of parallel computation:

(2) ∂Vm∂t(x_,t)=1Cm[−∑Iion(x_,t)−Iapp(x_,t)  +1β(kk+1)∇·(Di(x_)∇Vm(x_,t))],

where *I*_ion_ is the sum of all transmembrane ionic currents, *I*_app_ is the transmembrane stimulating current density, and *D*_*i*_ represents the diffusion coefficient.

In order to solve the monodomain equations, an initial equation (3) as well as boundary condition (4) were required:

(3) $$\begin{array}{c} V\left(\underline{x},t=0\right)=V\left(\underline{x}\right), \end{array}$$

(4) $$\begin{array}{c} n\cdot V\left(\underline{x},t=0\right)=0. \end{array}$$

#### 2.1.3. Electromechanical Coupling

In this study, we have modeled the electrical excitation and mechanical contraction as two separate processes weakly coupled together through the use of the excitation wavefront to drive the active tension development.

**(1) Myocardium Mechanical Properties**

After the cardiac electrical excitation sequences were determined by an excitation propagation algorithm in the electrical simulation, the resultant active forces of the myocardium were calculated. The active stress development in the myofibers depended on the time and sarcomere length and was initiated at the timing of the depolarization, as Kerekhoffs et al. [22–24] did. Then the active force along the fiber was calculated.

**(2) Finite Element Method (FEM)**

**Figure 3:**
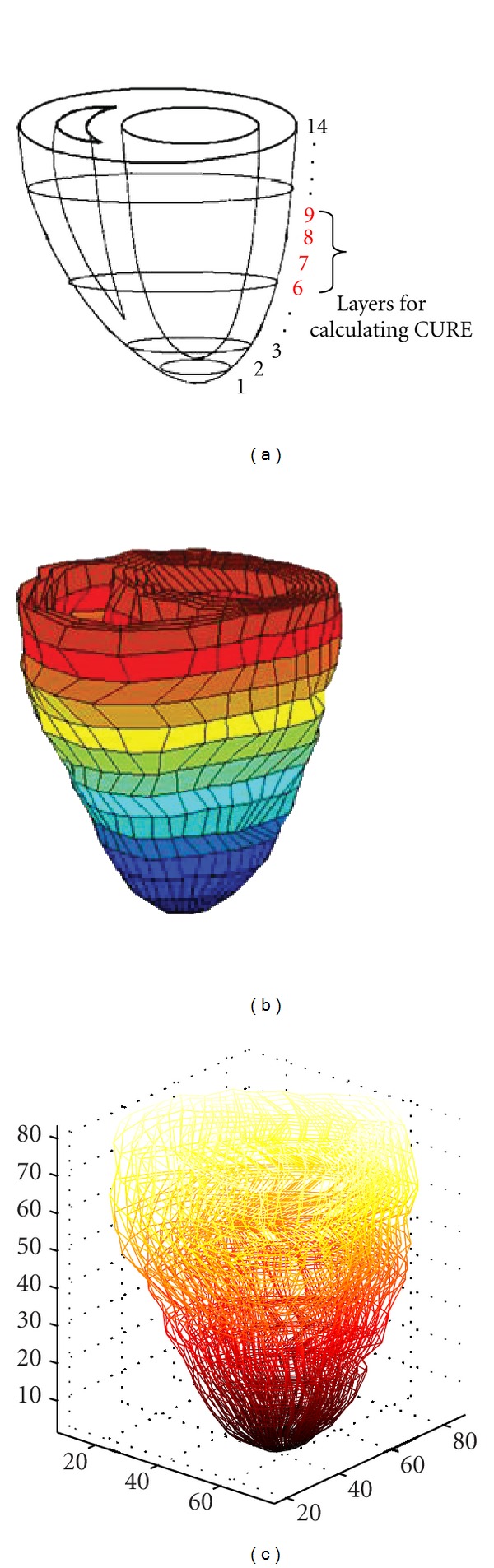
Schematic for layer display. (a) Four short slices near the equator site (layer 6 to layer 9) for calculation of CURE. (b) The finite element meshes of biventricular mechanical model of canine. (c) The coordinate system of biventricular mechanical model of canine.

In this work, the ventricular walls were divided into 14 layers from apex to base along the long axis of the LV (see Figure 3). There are in total 2,269 hexahedral elements and 8,736 degrees of freedom. A 3D 8-node isoparametric element is used as the basic element. Notice that the original reference heart geometry corresponds to the end-diastolic state of a dog heart. Also, muscle alike restriction was added to ventricular base elements due to the constraint of pleura.

To analyze the motion of ventricles, the following equation at time *t* need to be computed:

(5) $$\begin{array}{c} \left[K\right]\left\{\delta \right\}=\left\{F_{f}\right\}, \end{array}$$

where [*K*] is the total stiffness matrix, {*δ*} is the volume vector of nodal displacement, and {*F*_*f*_} is the total vector of the active forces:

(6) $$\begin{array}{c} K=\sum_{e}{\left[K\right]}^{e};\;\;F_{f}=\sum_{e}{\left[F_{f}\right]}^{e}, \end{array}$$

where [*F*_*f*_]^*e*^ is the nodal force vector of an element described above, and [*K*]^*e*^ is the stiffness matrix of an element with the following expression:

(7) $$\begin{array}{c} {\left[K\right]}^{e}=\sum_{l=1}^{L_{e}}\int_{\xi_{l-1}}^{\xi_{l}}\left(\sum_{m=1}^{M_{e}}\int_{\eta_{m-1}}^{\eta_{m}}\int_{-1}^{1}{\left[B\right]}^{T}{\left[C\right]}_{lm}\left[B\right]\left|J\right|d\xi d\eta \right)d\zeta , \end{array}$$

where [*C*]_*lm*_ is the elasticity matrix of layer *l* and segment *m*/*M*_*e*_ denotes the number*/*total numbers of segments in layer *l* in the circumferential direction, respectively. 

In the fiber-coordinate system, the nodal force vector of each element {*F*_*f*_}^*e*^ in the direction of fiber can be calculated as shown in the following:

(8) {Ff}e  =−∑l=1Le∫ξl−1ξl∬−11[B]TT{0,0,σe′,0,0,0}lT|J|dξdηdζ,

where [*B*] is the geometric matrix of an element, *σ*_*e*_′ is the active myofiber stress as a function of time after onset of contraction and sarcomere length history, |*J*| is the determinant of the Jacobin matrix, *ξ*,  *η*,  *ζ* the local coordinate system with the magnitudes ranging from −1 to 1, *l* and *L*_*e*_ are the number and total number of layers in an element, respectively, and *T* is the transformation matrix between the fiber coordinate and global coordinate (see Appendix A). 

These constitutive relations for the cardiac mechanics were then incorporated into a continuum electromechanical model of biventricles to predict the displacement and deformation. From (5) to (7), we can calculate displacement {*δ*}. The equations related to mechanics are solved with eight-node isoparametric element method. Local myocardial circumferential strain *ε*_*cc*_ of the biventricles is then calculated during the systole phase. As far as mechanical properties are concerned, the material was considered as transversely isotropic at any point in the myocardium. The in-vitro stiffness parameters and material constants were used as before [20].

### 2.2. Optimization Strategies 

#### 2.2.1. Mechanical Index

Mechanical dyssynchrony is a potential indicator for predicting responses to CRT. For example, novel echocardiographic image speckle tracking applied to routine midventricular short-axis images can quantify dyssynchrony by calculating the radial strain from the multiple circumferential points averaged to several standard segments and thus predict the response to CRT [25]. Dyssynchrony for the study was usually defined as the difference in the timing of the peak strain from the earliest to latest segment. However, most clinicians prefer to use the circumferential uniformity ratio estimate (CURE) as the assessment of the mechanical dyssynchrony in the practice of CRT [15, 26, 27]. Therefore, in this study, the CURE is used to index the mechanical dyssynchrony.

According to the report [15], short-axis slices motion near the equator can reflect the actual synchronous systolic process of the ventricular wall; thus, in this study, we chose four short-axis slices near the equator site (layers 6 to 9 in our heart model) to calculate CURE, excluding the most apical and basal regions (Figures 3(a), 3(b)). The coordinate system of biventricular mechanical model of canine was shown in Figure 3(c). The algorithm are as follows.

**Step 1**

**Figure 4:**
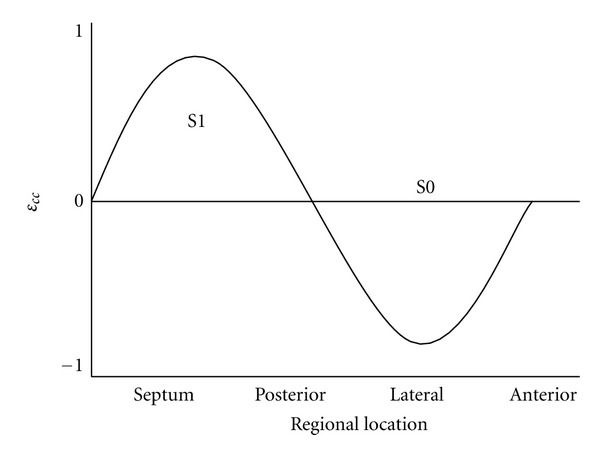
Circumferential strain *ε*_*cc*_ plotted as a function of spatial location of heart segment at a given time. Data were first processed by Fourier series decomposition. The zero-order (S0) and first-order (S1) terms shown plotted versus spatial position.

Compute circumferential strain *ε*_*cc*_ over the entire LV-midwall (*y*-axis) at 30 circumferentially-distributed locations around each short-axis section (*x*-axis), and plot *ε*_*cc*_ versus spatial position for each time-frame. The more oscillatory the plot, the greater was the dyssynchrony among the segments around the short axis. To explain this, we can plot circumferential strain versus spatial location of the segment as Figure 4 showed. Then, from the data, zero-order S0 and first-order S1 terms can be obtained by Fourier series decomposition. A perfectly synchronous heart appeared as a straight line (solely S0 term), whereas one that was perfectly dyssynchronous would appear as a sinusoid (S1 term) (Figure 4).

**Step 2**

Plots for the four mid-wall short-axis slices are then subjected to Fourier analysis with a function of fft() in Matlab7.0.1, and the results are averaged over space and time to yield

(9) $$\begin{array}{c} \text{CURE}={\left(\frac{A_{0}^{2}}{A_{0}^{2}+2A_{1}^{2}}\right)}^{1/2}, \end{array}$$

where *A*_0_^2^ and *A*_1_^2^ are the spatial and temporal sums of the zero- and first-order power terms, respectively. The ratio of mean to “mean plus first-order power” provides the CURE index, and the maximal value for CURE is 1 with all segments contracting synchronously; whereas symmetrically dyssynchronous contractions produce a CURE = 0 [15, 26, 27].

A further description of calculating zero and first-order power terms of layer 6 to 9 around equator sites at a given time was given in Appendix B. Then with (9), CURE can be obtained.

**Step 3**

Compute the CURE for each electrode pair and each VVD and choose maximum CURE as optimal result of CRT. Because the left ventricular dyssynchrony is an important determinant of CRT response, the CURE calculated from LV and septum is chosen alternatively as the criterion for predicting the response to CRT in the simulation.

The optimization method was then applied to the electromechanical canine heart model of LBBB. The optimization parameter of the CURE was sequentially calculated for each combination of the pacing location and VVD. The optimal result of CRT can be determined by the combination that provided the maximum CURE. 

#### 2.2.2. Electrical Index

The description of the electrical optimization strategy with *E*_RMS_ can be found in many published reports. Briefly, it is assumed that the optimal cardiac output is to be achieved through the sinus rhythm and the normal electrophysiological parameters [16]. Therefore, the aim of the pacing therapy is to restore an electrophysiological status as close as possible to the physiological electromechanical coupling. Here, we calculate the root mean square error *E*_RMS_ between the physiological excitation during the sinus rhythm and pathological excitation as below:

(10) $$\begin{array}{c} E_{\text{RMS}}=\sqrt{\frac{1}{N}\sum_{i=1}^{N}{\left(x_{i}-e_{i}\right)}^{2}}, \end{array}$$

where *N* is the whole number of voxel elements, *x*_*i*_ dedicates the activation time of voxel *i* under the sinus rhythm, and *e*_*i*_ is the activation time of voxel *i* under the pathological condition. Thus, by minimizing the *E*_RMS_, the activation of the heart will be as close as possible to the healthy activation and will provide an optimal combination of the pacing site and timing delay in CRT. 

### 2.3. Objective Function of the Optimization Problem

In order to make a contrast analysis of cardiac function improvement between mechanical (CURE) and electrical (*E*_RMS_) optimization strategy for CRT, two objective function are defined as below.

#### 2.3.1. Dyssynchrony Index (DI)

Dyssynchrony for the study was usually defined as the difference in the timing of the peak strain from the earliest to latest segment, as represented by DI, which can be used to describe the contraction synchrony of biventricles. In the clinic research, the radial strain was usually used to substitute for the maximum principal strain. However, they all indicated a thickening of cardiac walls.

#### 2.3.2. Ejection Fraction (EF)

The hemodynamic parameter of EF, which is the percentage of blood ejected from the ventricle with each heartbeat, can determine the cardiac function. Since LV is the heart's main pumping chamber, EF is usually measured only in the LV. A measure of the function of LV, also called left ventricular ejection fraction (LVEF), can help determine the cardiac hemodynamics effect. LVEF is defined as

(11) $$\begin{array}{c} \text{LVEF}=\frac{\left(\text{EDV}-\text{ESV}\right)}{\text{EDV}}, \end{array}$$

where EDV means LV volume of end-diastole while ESV represents the LV volume of end-systole.

### 2.4. Pacing Site and VVD

#### 2.4.1. Pacing Site

**Figure 5:**
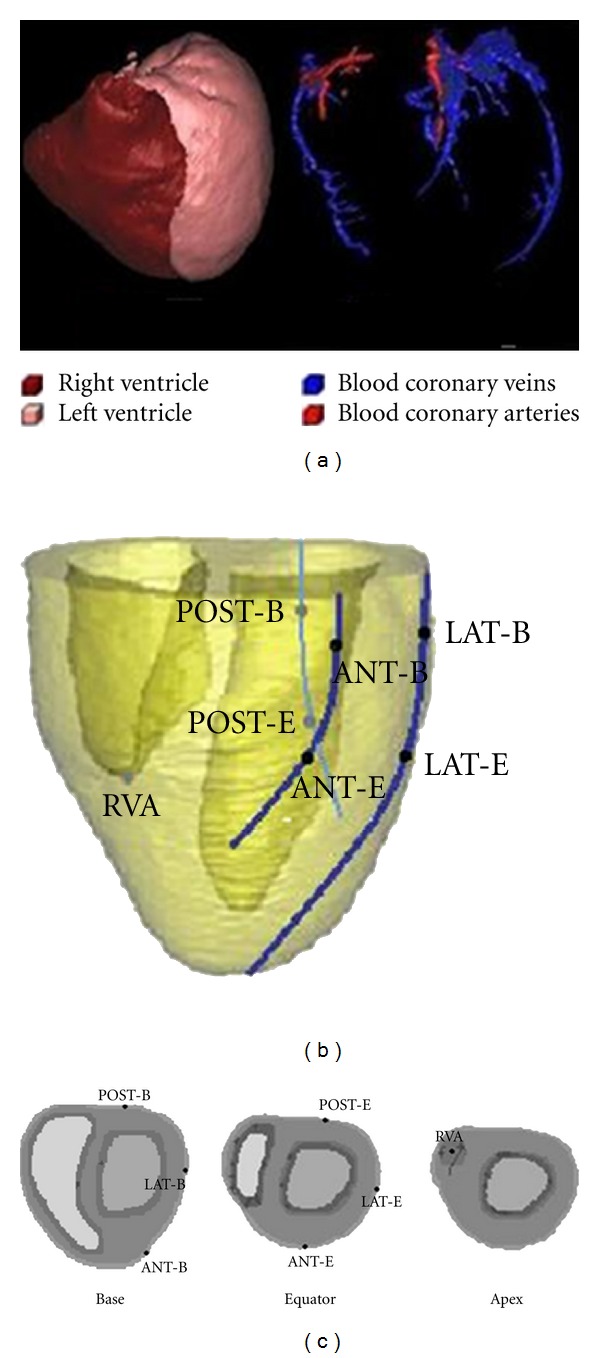
A diagram of the electrode positions of pacing leads. (a) Reconstructed LV, RV, blood coronary veins (blue) and blood coronary arteries (red) from USA VHP female dataset. (b) Three-dimensional display of LV and RV pacing lead positions. (c) Display of pacing lead positions on transverse plane.

In this study, BiVP was chosen for CRT simulations. In clinic, doctors often choose the branches of coronary sinus as the LV pacing lead site as shown in Figure 5(a). Here, the anterior, lateral, and posterior branches of the coronary sinus were plotted according to the original anatomical data as shown in Figures 1(c), 1(d). Moreover, to better contrast the influence of the different LV pacing lead positions to the response of CRT, theoptional LVpacing sites were chosen at the base and equator sites of the LV along the branches of the blood coronary veins, as shown in Figure 5(b). The pacing lead positions on the transverse plane are shown in Figure 5(c). The right ventricular apex (RVA) of the endocardium, point RVA (36, 79, 49), was chosen as the position of RV pacing lead. The LV electrodes were placed in the anterior and posterior wall of the LV in addition to the LV free wall (LVFW) as followed, POST-B (77, 98, 89) at the posterior base, POST-E (79, 94, 57) at the posterior equator, LAT-B (114, 62, 89) at the lateral base, LAT-E (108, 53, 57) at the lateral equator, ANT-B (90, 15, 89) at the anterior base, and ANT-E (67, 20, 57) at the anterior equator. Then, pacing was performed for each pair of right and left ventricular pacing positions yielding six electrode setups for the CLBBB model to investigate the influence of the electrode position on the response to CRT. 

#### 2.4.2. Pacing Timing Delays

Generally, the activation of the sinus node serves as the reference time for the AV delay. Several studies have pointed out that the VVD should be optimized according to the changes in the AV delay to obtain the maximum hemodynamic benefit [16]. However, because the original canine data did not include the atria, the construction of a whole heart model was infeasible and therefore the AV delay was kept constant in this research. Considering the special features of the canine activation period, we choose −72 ms, −60 ms, −48 ms, −36 ms, −24 ms, −12 ms, 0 ms, 12 ms, 24 ms, 36 ms, 48 ms, 60 ms, and 72 ms as the VVD timings. To contrast with clinic studies, the scope of VVD timings was enlarged to better investigate the influence of VVD on the optimal efficacy of CRT. Positive values indicated that the LV was the first ventricle stimulated and a negative VVD indicated a right-before-left ventricular stimulation. Therefore, there were 78 simulations in whole by multiplying pacing leads numbers and VVD numbers. So, an optimization can be achieved by maximizing the CURE or minimizing the *E*_RMS_ through adjustment of the pacing location and VVD.

### 2.5. Numerical Solutions

Simulation of the cardiac excitation anisotropic propagation throughout the ventricular myocardium is computationally very expensive. Thus, high-performance computing techniques should be used. By using an operator-splitting scheme, adaptive time step, and backward differentiation formulation techniques in a parallel implement, we solved the monodomain equations of the cardiac excitation anisotropic propagation successfully [28]. For a combination of the pacing location and VVD, the model of the electrical propagation ran for approximately 10 hours on a Dell computer with four 3.0 GHz Xeon processors running in parallel and with 4 GB of RAM. The computation of the CURE took approximately 190 MB of main memory and 1.5 hours on a Dell computer with a single 3.0 GHz Xeon processor.

## 3. Results

### 3.1. The Optimal Result of CRT

**Figure 6:**
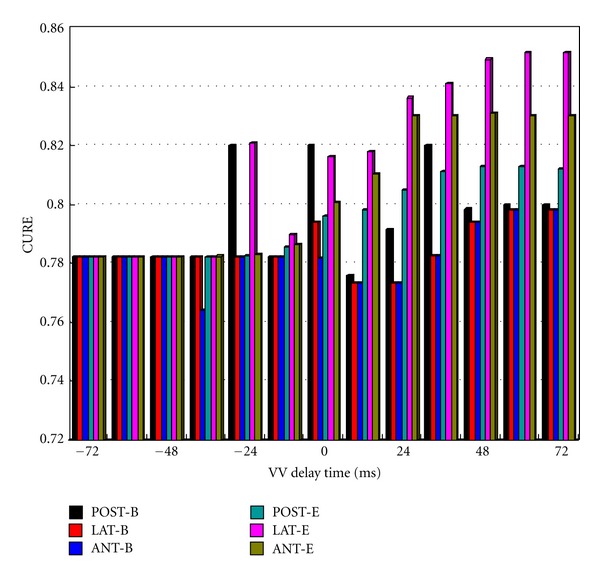
A column map of CURE-VV delay time curve.

The maximal value of CURE was 0.8516, with pacing site at point LAT-E and a VVD of 60 ms (see Figure 6), which could be regarded as the optimal result for CRT. The calculated CURE value of the LBBB model was 0.67. According to clinical reports, cardiac mechanical synchrony was also less in patients with LBBB (CURE in one cardiac cycle: 0.58 ± 0.09) [27]. The value of the maximum CURE significantly increased after BiVP optimization, indicating that CRT could enhance the intrasynchrony of LV for CHF hearts with LBBB. 

With the electrical optimization strategy, an optimal pacing location and VVD were also found with a minimal error *E*_RMS_ of 37.26 ms. The optimal stimulation site was also at point LAT-E, but with a VVD of 0 ms. 

To find the potential relationship between CURE and VVD, a diagram of the CURE-VV delay time column map was plotted in Figure 6. It was found that the values of CURE were larger when VVD ≥ 0 ms, which meant LV was the first stimulated. But for different LV pacing sites, CURE varied disorderly without a continued trend of ascending or descending with the change of VVD. It indicated that there existed a nonlinear relationship between CURE and VVD time. However, with the increasing of VVD, especially when VVD ≥ 48 ms, CURE varied little and the same thing happened when VVD ≤ −48 ms. For VVD ≥ 60 ms, CURE would not rise again. It was obvious that CURE was not the largest with VVD = 0 ms that commonly used in the previous VVD setup.

Furthermore, CURE values of LV pacing sites near ventricular equator sites were all larger than those at base for VVD ≥ 12 ms. However, the CURE of LV pacing location near the lateral wall at equator site, that is, pacing location LAT-E, was always the largest. It can also be found that the CURE values were smaller when the pacing lead was at the anterior wall near base, that is, pacing location ANT-B and ANT-E. 

### 3.2. Optimal Result Contrast of CRT between CURE and *E*_RMS_

**Figure 7:**
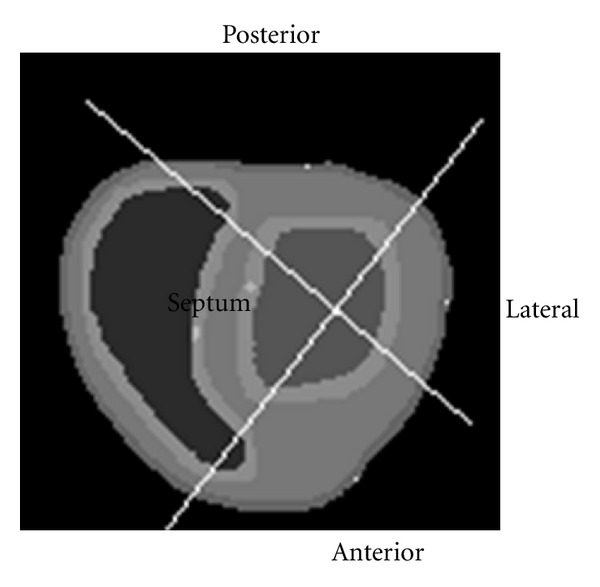
Segments division of the short-axis LV, named anterior wall, lateral wall, posterior wall, and septum.

**Figure 8:**
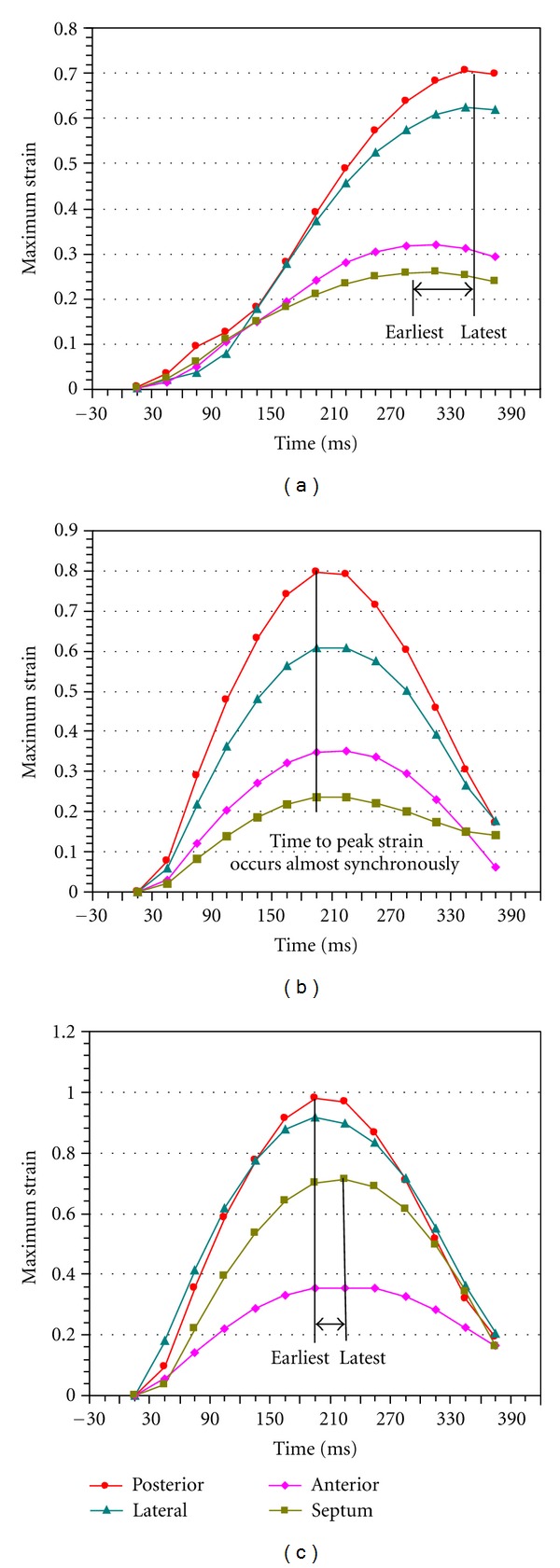
An example of maximum principal strain-time curves. (a) a CHF study model with LBBB. (b) a CRT optimization model with maximum CURE. (c) a CRT optimization model with minimum *E*_RMS_. The curves are color-coded by the defined myocardial regions as depicted in the figure. An example of dys-synchrony is shown as the difference in the timing of the peak strain from earliest to latest segment (black arrow). (a) shows that DI equals to 60 ms. (b) shows that four segments arrive at the peak-of-strain almost at the same time. (c) shows that DI equals 30 ms.

In order to better contrast with clinical findings, we divided the short-axis LV into four segments, named anterior wall, lateral wall, posterior wall, and septum (Figure 7). Then, the positive maximum principal strain (also called maximum strain) was calculated from the multiple circumferential points averaged to several standard segments near the equator sites (layer 7). Maximum strain was plotted against time under three conditions, including a CHF model with LBBB, a CRT optimization model with maximum CURE and a CRT optimization model with minimum *E*_RMS_ (Figure 8), and thus predicted their responses to CRT [25]. The maximum strain,*E*1, was used to indicate thickening of the cardiac walls [20]. *E*1 is the maximal eigenvalue of the Green Lagrange strain tensor *E* with the expression of

(12) $$\begin{array}{c} E=\frac{1}{2\left(F^{T}F-I\right)}, \end{array}$$

where *I* represents the identity matrix and the superscript *T* represents the matrix transpose, and deformation gradient tensor *F* indicates both the rotation and the deformation around a point. 

In the simulation of LBBB, as shown in Figure 8(a), DI equals 60 ms with septum the earliest to the peak strain while lateral and posterior wall the latest to the peak strain. LVEF in the simulation of LBBB is 22%. However, in the simulation of CRT with mechanical optimization strategy (CURE = 0.8516), the time for all four segments (anterior wall, lateral wall, posterior wall, and septum) to peak strain occurs almost simultaneously, as shown in Figure 8(b), and therefore DI equals 0 ms. LVEF in the simulation of BiVP is 35%, which means an enhancement of LV hemodynamic function after CRT optimization with mechanical strategy.

The “Maximum Strain-Time” curve with the minimum *E*_RMS_ (37.26 ms) was plotted as the optimal result of CRT from the electrical index (Figure 8(c)). Contrary to the LBBB result, lateral and posterior wall were the earliest to peak strain while septum was the latest to peak strain. The optimal pacing lead was located at the point LAT-E with a VVD of 0 ms. The calculated DI was 30 ms with a LVEF of 30%.

## 4. Discussions

The clinical findings of CRT have demonstrated that the adjustment of pacing lead position and VVD were very important for an individual to get the best improvement in hemodynamics [29]. However, there still need more quantitative information for determining the optimal VV timing pre- or postoperatively with respect to the lead positions in patients undergoing CRT. Based on a coupled biventricular electromechanical model of the canine with CLBBB, the mechanical and electrical optimization strategies for predicting the effect of CRT were investigated. Unlike previous electrical optimization strategy of CRT (*E*_RMS_), we have adopted the mechanical optimization strategy CURE as the synchrony assessment index in the research. As we know, the main function of heart is to pump blood and exchange the metabolites. Therefore, the mechanical function of heart and the cardiac hemodynamics were the most basic characteristics and essential criteria for evaluating the cardiac function.

The implantation location of LV pacing lead was proven to be very important for the improvement of the ventricular systolic synchrony [30]. From Figure 6, it indicated that the optimal pacing location was at the LV lateral wall (LVLW) near the equator site (point LAT-E) based on the mechanical index of CURE. It can also be found that a larger CURE could be obtained by placing stimuli at the posterior wall at base (point POST-B) or at the anterior wall near equator sites (point ANT-E). It was suggested by clinicians that the pacing lead should be implanted in the LV posterior or the lateral vein of the coronary sinus during the BiVP in accordance with our simulation results [30]. Thus, the mechanical synchronous contraction of the myocardium was forced to be better. According to clinical researches [31, 32], the left ventricular posterior wall (LVPW) and LVLW were often the latest activation sites in LBBB patients. In other words, we also validated that the latest activation or mechanical contracting site may be the optimal stimulating site, which was also validated by the mathematical model of Helm et al. [33]. Furthermore, Figure 6 also showed us that a better LV systolic synchrony (LVSS) could be achieved by placing pacing electrodes near the equator sites (points POST-E, LAT-E, and ANT-E) than near the base sites (point POST-B, LAT-B, and ANT-B).

However, with the increasing of VVD, especially for VVD ≥ 48 ms, CURE varied little and the same thing happened when VVD ≤ −48 ms. For VVD ≥ 48 ms, LV was the first ventricle to be paced. However, when RV was paced, the intrinsic excitation or excitation from LV pacing lead had spread to the RV beforehand. Due to the influence of refractory period, the pacing lead of RV (point RVA) would not play a role. Therefore, CURE values make no variations when VVD was larger than a certain value.

From Figure 6, it showed us that all maximal CURE values were larger than 0.8150 when VVD ≥ 0 ms. We could also find that a larger CURE may be obtained when the VVD equals 0 ms, especially for those with the LV pacing leads located at the equator sites. It has been observed by clinicians that a larger CURE might be obtained when the LV was first stimulated (VVD ≥ 0 ms) and about 28.4% of patients might get optimum hemodynamics when a VVD of 0 ms was offered [34]. Under pathological conditions of LBBB, normal rhythm was broken up and depolarization spread more slowly and less uniformly. Due to the spoiled excitation propagation, there was a profound abnormality of contraction sequences of both ventricles, which affected ventricular contractile activity and decreased the heart function. During simulation of LBBB (Figure 2), electrical and mechanical delay was evident in the LVFW segments, which suggested a severe interventricular dyssynchrony [20]. Therefore, LV needs to be paced first to get a coordinate contraction of whole heart during CRT. Before the invention of the second-generation dual-chamber pacemaker, the initial factory setting for the VVD was 0 ms. However, it could be found from our simulation results that the optimal VVD was not 0 ms but 60 ms, based on the mechanical strategy of predicting the response of CRT. Therefore, it will still be necessary for the optimization of VVD. 

To further observe the enhancement of LV synchronization after CRT optimization and to contrast the optimal result between mechanical (CURE) and electrical (*E*_RMS_) strategy, Maximum Strain-Time curves were plotted in Figure 8. In the simulation of LBBB (see Figure 8(a)), the time to peak strain of all the ventricular segments had various degrees of delays in accordance with the clinic phenomenon [30], with a DI of 60 ms and a LVEF of 22%. Nevertheless, the optimal results with the CURE indicated that LV had a better synchrony (DI = 0 ms, meaning that all the LV segments contract almost simultaneously, as shown in Figure 8(b)) and a higher LVEF (35%), whereas the optimal result of CRT from the electrical criterion (*E*_RMS_) had a worse synchrony (DI = 30 ms, meaning that the LV still has some dys-synchrony, as shown in Figure 8(c)) and a lower LVEF (30%). The results proved that mechanical index (CURE) used in this model was superior to the electrical index (*E*_RMS_) for the optimal parameters of CRT. It also confirmed the fact that the CURE could better reflect the LV synchrony and hemodynamic result, although biventricles may not obtain the best electrical synchrony [25]. It has been proven by doctors that systolic improvement and mechanical resynchronization does not require electrical synchrony in the dilated failing heart with LBBB [15]. Mechanical coordination plays the dominant role in the global systolic improvement with the BiVP approach. Besides, it may be easier for us to optimize the parameters of CRT using a mechanical criterion with clinic image methods, such as MRI, intracardiac ultrasound, and so on. Several studies have suggested that the measures of mechanical dys-synchrony by cardiac imaging were superior markers of the response to CRT, compared with the ECG QRS duration [25, 27, 33, 35]. In the practice of CRT optimization, clinicians often use the TDI to trace “tissue velocity-time” with the septal-to-lateral delay of the maximum velocity defined as the LV dyssynchrony, or use echocardiograph techniques to trace “radial strain-time” curves with ealiest to latest delay of peak strain defined as the LV dyssynchrony. The VVD that could produce the smallest septal-to-lateral delay and the maximum cardiac output (CO) would be selected as the optimal VVD of CRT, similar to our validation methods [25]. However, note that in this research, improved mechanical coordination and pump function may be observed in the optimal results (DI = 60 ms, LVEF = 22%) with both the CURE and *E*_RMS_ as optimization strategy for CRT. The thickening of the cardiac walls also improved after CRT with the peak maximum principle strain (PMPS) increased from 0.70 in the LBBB model to 0.77 in CRT optimization model based on the CURE strategy as shown in Figure 8(c). However, the PMPS in CRT optimization model based on the *E*_RMS_ strategy was larger, with a value of 0.98 as shown in Figure 8(c), indicating a better local myocardium contraction function.

Previous electrical optimization procedures were based on the electrical isochrones, that is, the electrical activation of the cells, which did not necessarily equal the electromechanical coupling, that is, the phenomenon of the delayed electrical uncoupling. Assuming that the mechanical activation followed the electrical activation with a fixed delay, the results with the electrical optimization strategy of CRT might be valid, because the timing offset would be added to all cardiac cells. So long as the delay between the electrical and the mechanical activation per cell varied, the electrical optimization strategy of CRT might be not accurate, because of the pathological prolongation of the mechanical activation influenced by the delayed electrical uncoupling. Damage to the myocardium intercalated disks was another phenomenon of electromechanical dissociation. Because the conduction of the electrical excitation was accomplished by the intercellular “gap junctional communication”, the path of the connection could be closed partly or completely when there was a very low blood calcium level or acidosis causing conduction disturbance in the damaged regions. For a heart with an electromechanical dissociation, the adjustment of the pacing location or VVD might produce profitable results, but the optimization of the pacing site and VVD of CRT with the electrical optimization strategy might be given a great discount [18]. 

Briefly, the electrical and mechanical activations were not completely equal to each other. However, with the mechanical criterion (CURE or other hemodynamical index) to optimize the parameters of CRT, the problem could be solved. Alternatively, an improved mechanical coordination and function may be inducible in the LBBB-CHF hearts without generating electrical synchrony. The difference of optimal delays and pacing sites was due to the difference of heart geometry, electrical and mechanical properties, and also the time regional differences in the time between electrical excitation and mechanical contraction at one part of the model versus another part. Therefore, clinicians would not always choose the lateral wall for the pacing locations. With the help of a patient-specific computation heart model, quantitative information will be obtained to enhance our understanding of cardiac dyssynchrony and decrease the blindness of CRT. 

### 4.1. Limitations

In this study, however, there are still some defects in our optimization model of CRT. Because of the lack of the atria data, the construction of a whole heart model was not feasible and consequently, the adjustment of the AV delay has not been considered. Furthermore, the hemodynamics parameters should be taken into account in future modeling, such as the cardiac output and the *dP*_max⁡_/*dt* (maximum value for the first derivative of the LV pressure (peak *dP*/*dt*)) that were usually used as the optimal parameters of CRT in clinic [25, 36]. In addition, the heavy computation load might limit the clinical applications of the computation model. Another major limitation of this study is the use of a standard heart model. However, in clinical practice, there are numerous variations, for example, various geometry of the heart, various electrical properties of the heart (conduction velocities, conduction blocks, infarct zones, etc.), various mechanical properties of the heart, various autonomic tones, and so on. Therefore, the results of the single simulation study cannot be extrapolated to clinical patients now.

## 5. Conclusion

By using a coupled biventricular electromechanical models of the canine heart, the optimization of the electrode positions and VV delay timings for CRT with the BiVP have been investigated,  with both the mechanical criterion of CURE and electrical criterion of  *E*_RMS_. We also demonstrated that the mechanical dyssynchrony measure was able to predicts well the effect of CRT, better than the electrical dyssynchrony measure.

In comparison with other computational optimization models of CRT, the mechanical contraction and deformation was included in our model, and the mechanical index CURE was calculated. The results indicated that the LV pacing lead positioning was a very important factor that affecting the consequence of CRT and the hemodynamic changes. Also, the changes of VVD will lead to a variation in the mechanical synchrony and should be considered in the optimization of CRT. Therefore, it was very important for an individual to adjust the electrode position as well as the timing delays to the patient's anatomy and pathology, in accordance with current clinical studies [34, 37, 38]. In addition, we can conclude from simulation results that the site of the latest mechanical activation may be the optimal left ventricular lead position for the current LBBB-HF model, but it may be limited by the anatomy of the coronary vein. Compared with the electrical optimization strategy, the simulation results showed that the LV synchronous contraction and hemodynamics could be improved more with the mechanical optimization strategy for predicting the effect of CRT. Mechanical dyssynchrony, rather than the electrical dispersion, seems to be the more relevant. Therefore, mechanical dyssynchrony is a potential better means for predicting the response to CRT. However, it does point out that, to apply this modeling approach in clinical practice, patient-specific electromechanical heart model must be established [39].

## Figures and Tables

**Figure 9:**
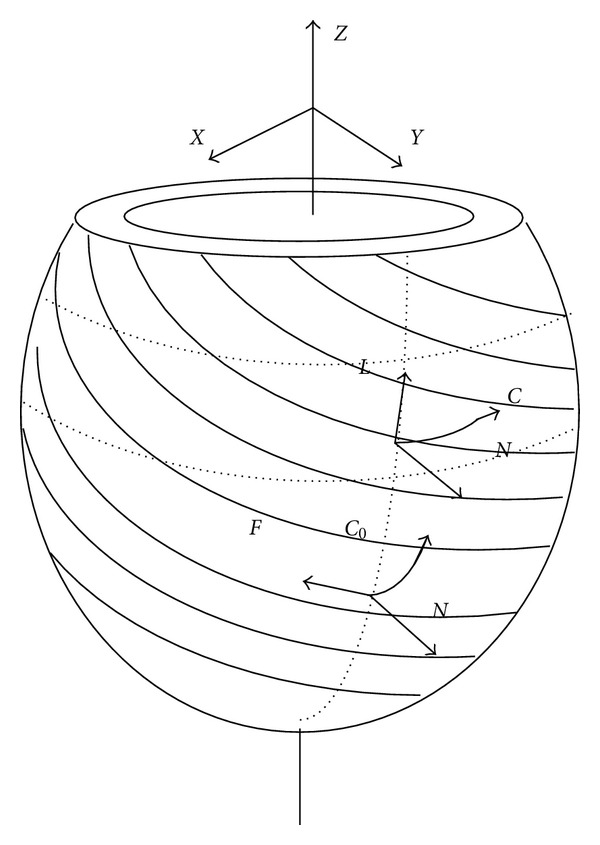
Cardiac walls coordinate systems.

## Appendices

### A. Calculation of the Transformation Matrix between the Fiber Coordinate and Global Coordinate

Since fiber orientations vary for different sublayers in an isoparametric element of hexahedron, the transformation between the fiber coordinate and the global coordinate is needed. As shown in Figure 9, we first need to transfer the fiber-coordinate system (*N*-*C*_0_-*F*, one axis is chosen to coincide with the local muscle fiber direction *F*, and another one is determined by the epicardium surface normal vector *N*) to the new surface-coordinate system (*N*-*C*-*L*, one axis is chosen to coincide with the epicardium surface normal vector *N*, and another one is determined by the epicardium circumferential or tangential vector *C*), with the transformation matrix *T*_1_ determined by the fiber direction angle of a single sublayer; then transfer the surface-coordinate system (*N*-*C*-*L*) to the global coordinate system (*X*-*Y*-*Z*), with the transformation matrix *T*_2_ determined by the direction cosines between two doordinate systems.

Finally, the transformation matrix *T* between the fiber coordinate and the global coordinate is as follows:

(A.1) $$\begin{array}{c} T=T_{2}T_{1}. \end{array}$$

### B. Calculation of Zero and First-Order Power Terms

The codes for calculating zero and first-order power terms of layer 6 at a given time in Matlab7.0.1 are as follows:

(B.1) $$\begin{array}{c} y=\text{Sheet}6\left(1,:\right);\\ FY=\text{fft}\left(y\right);\\ \text{mag}=\text{sqrt}\left(FY.*\text{conj}\left(FY\right)\right);\\ \text{s}02\_6=\text{mag}\left(1\right);\\ \text{s}12\_6=\text{mag}\left(2\right); \end{array}$$

where Sheet6(1, :) are the circumferential strains (*ε*_*cc*_) over the entire LV-midwall at 30 circumferentially distributed locations in layer 6.

From above, we can get the zero power term (s02_6) and first-order power term (s12_6) of layer 6 at a given time. Then the zero and first-order power terms of layer 7 to 9 during the cycle can be obtained in the same way.
